# Tracheostomy in Severe Bronchopulmonary Dysplasia—How to Decide in the Absence of Evidence

**DOI:** 10.3390/biomedicines11092572

**Published:** 2023-09-19

**Authors:** Audrey N. Miller, Edward G. Shepherd, Amy Manning, Humra Shamim, Tendy Chiang, George El-Ferzli, Leif D. Nelin

**Affiliations:** 1 Comprehensive Center for Bronchopulmonary Dysplasia, Department of Pediatrics, Division of Neonatology, Nationwide Children’s Hospital, Ohio State University College of Medicine, Columbus, OH 43205, USA; audrey.miller@nationwidechildrens.org (A.N.M.); edward.shepherd@nationwidechildrens.org (E.G.S.); george.el-ferzli@nationwidechildrens.org (G.E.-F.); 2 Department of Otolaryngology, Nationwide Children’s Hospital, Ohio State University College of Medicine, Columbus, OH 43205, USA; amy.manning@nationwidechildrens.org (A.M.); humra.shamim@nationwidechildrens.org (H.S.); tendy.chiang@nationwidechildrens.org (T.C.)

**Keywords:** bronchopulmonary dysplasia, tracheostomy, mortality, pulmonary outcomes

## Abstract

Infants with the most severe forms of bronchopulmonary dysplasia (BPD) may require long-term invasive positive pressure ventilation for survival, therefore necessitating tracheostomy. Although life-saving, tracheostomy has also been associated with high mortality, postoperative complications, high readmission rates, neurodevelopmental impairment, and significant caregiver burden, making it a highly complex and challenging decision. However, for some infants tracheostomy may be necessary for survival and the only way to facilitate a timely and safe transition home. The specific indications for tracheostomy and the timing of the procedure in infants with severe BPD are currently unknown. Hence, centers and clinicians display broad variations in practice with regard to tracheostomy, which presents barriers to designing evidence-generating studies and establishing a consensus approach. As the incidence of severe BPD continues to rise, the question remains, how do we decide on tracheostomy to provide optimal outcomes for these patients?

## 1. Introduction

The incidence of bronchopulmonary dysplasia (BPD) continues to increase, likely secondary to the improved survival of extremely preterm infants [1]. The most severely affected subset of infants with BPD are classified as having grade 3 BPD (also called type 2 severe BPD), which is characterized by the ongoing need for invasive positive pressure ventilation (IPPV) at 36 weeks postmenstrual age (PMA) [2,3]. Infants with grade 3 BPD are six times more likely to need a tracheostomy for chronic ventilation compared to those with grade 2 BPD, which is defined as the need for non-invasive positive pressure (i.e., nasal constant positive airway pressure (CPAP), etc.) at 36 weeks PMA [4]. Tracheostomy is usually considered in infants that are of term-corrected age or older, unable to wean from IPPV within a reasonable time-frame, and/or have had multiple unsuccessful weaning attempts.

Severe BPD is the most common reason for tracheostomy placement in infants [5]. Furthermore, the proportion of infants and children with BPD who are dependent on positive pressure via tracheostomy has increased over time [6,7,8]. The BPD Collaborative recently reported using their registry data that 23% of a cohort of 524 patients with severe BPD had undergone tracheostomy [4]. It has been estimated that in the United States, at least 200 infants with severe BPD are discharged on home mechanical ventilation annually, with an estimated 2000 children with severe BPD on home ventilation via tracheostomy at any one time [9].

**Figure 1:**
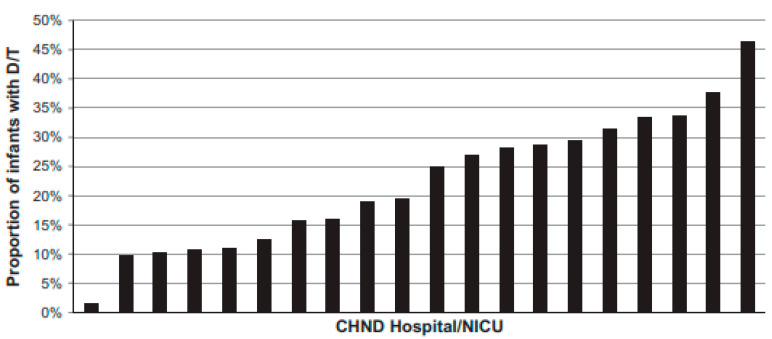
Inter-center variation in death or tracheostomy placement (D/T). Reused with permission from Springer Nature [10].

Interestingly, there is substantial variation among centers regarding tracheostomy placement, as in Figure 1 from reference [10]. One sizeable multicenter study revealed that center was an independent risk factor for tracheostomy [10]. Additionally, there is significant variation by center regarding the median age at tracheostomy placement, initial hospital discharge, first outpatient visit, and decannulation [6]. This variation likely reflects the absence of evidence generated from high-quality trials to guide clinical decision making in infants with severe BPD, which further complicates the already difficult decisions surrounding tracheostomy. This review aims to summarize the best available information regarding tracheostomy outcomes, indications, timing, and long-term management to begin to provide a more universal framework for clinical decision making regarding tracheostomy in severe BPD in the absence of high-quality evidence.

## 2. Outcomes following Tracheostomy

### 2.1. Mortality

The question most important to parents when discussing tracheostomy is “will my child survive?” by which they mean, “what are the chances that my child survives into adulthood and beyond, like other children?” Unfortunately, this question is surprisingly difficult to answer. Published data are limited regarding the mortality rates of infants with BPD and tracheostomy, and these limited data report highly variable mortality rates. Furthermore, it is difficult to quantify the percentage of infants with severe BPD that die prior to tracheotomy placement; one study reported that 7% of infants with severe BPD died prior to tracheostomy placement [10]. Some studies have reported the mortality rates from the time of tracheostomy to the time of initial hospital discharge with a range of 9–23% [11,12]. Other studies report a combined mortality rate from the time of tracheostomy placement to a specified amount of time in the outpatient setting, and the available evidence on mortality rates in patients that received a tracheostomy for BPD is described in Table 1 [6,10,11,12,13,14,15,16,17,18,19,20,21,22]. In summary, the answer to the question “what are the chances that my child will survive into adulthood and beyond, like other children?” seems to be that there is a chance somewhere from 74% to 93% that a baby who receives a tracheostomy for BPD will survive their initial hospitalization and childhood.

BPD-associated pulmonary hypertension (BPD-PH), prematurity, small for gestational age status, and tracheostomy placement before one year of age have all been identified as risk factors for mortality in patients with severe BPD who have a tracheostomy [15,23]. One study examined the impact of mean airway pressure and fraction of inspired oxygen (FiO_2_) needed at the time of tracheostomy placement, and neither was associated with an increased risk for mortality [15]. The reported causes of death include tracheostomy complications (accidental decannulation, tracheal obstruction, mucous plugging of the tube), and those related to progression or severity of co-morbid conditions.

### 2.2. Respiratory Outcomes

Several studies [6,7,10,12,14,15,16] report median ages for tracheostomy placement, age for discontinuation of positive pressure, and age for decannulation (summarized in Table 2). In one study, 97% of surviving patients were liberated from positive pressure ventilation by five years of age, and those unable to do so were unlikely to ever achieve this milestone [7]. Other studies have examined longer-term respiratory outcomes for infants with BPD and tracheostomy. For example, one study examined childhood respiratory outcomes of patients with severe BPD both with and without tracheostomy and found lower childhood pulmonary function testing results in the tracheostomy group [24]. This study reported that patients with tracheostomy had a significantly lower mean childhood best forced expiratory volume in one second (FEV1) and mean FEV1/forced vital capacity (FVC) compared to infants with severe BPD without tracheostomy [24]. Another study also showed decreased pulmonary function in children with a history of tracheostomy, with spirometry measurements showing evidence of obstruction and airflow limitation [25]. The caveat of these findings is that they likely reflect the degree of overall BPD severity (i.e., only the patients with the most severe forms of severe BPD get tracheostomies) rather than any impact of the tracheostomy placement itself, thus, further studies are needed. One type of longer range follow-up study that might shed light on any potential effect of tracheostomy placement on lung function may be to determine if pulmonary function returns to levels seen in age-matched non-tracheostomized patients with BPD once the patients are decannulated.

### 2.3. Readmission

As is true for most technology dependent patients, re-admission rates are high in tracheostomy-dependent BPD patients. For example, 73% of infants with BPD and tracheostomy in one multicenter cohort required hospital re-admission for respiratory reasons within the first 12 months of initial hospital discharge [6]. Ehrenkranz et al. [26] found that the hospital re-admission rate for all infants with severe BPD was 39%, while Jensen et al. [2] reported that 29% of infants with grade 3 BPD had 2 or more hospitalizations for respiratory reasons. The most common reasons for re-admission for infants with BPD and a tracheostomy include respiratory infections and tracheostomy-related complications [27,28]. Importantly, the incidence of re-hospitalization decreases after decannulation [7], likely related to the elimination of device-related (tracheostomy tube) disease.

### 2.4. Tracheostomy Complications

Tracheostomy complications can be categorized as early (post-operative days (POD) 0–7) and late (POD > 7). The early period is critical for appropriate maturation of the stoma. Early complications are primarily related to immaturity of the stoma and include posterior tracheal wall tear, accidental decannulation, false passage, pneumothorax, subcutaneous emphysema, bleeding, infection, and skin breakdown [5,29]. In practice, the first tracheostomy tube change often marks the transition from the early to the late post-operative period with confirmation of the maturation of the stoma and ability to resume routine care. Late complications include cellulitis, tracheitis, tracheo-innominate fistula, mucus plugging, airway obstruction, and granulation tissue formation [5]. Some infants will develop chronic physiologic nasal congestion, laryngopharyngeal reflux, and Eustachian tube dysfunction secondary to a decreased nasal airflow and lymphoid hyperplasia [5].

### 2.5. Morbidities

Infants with severe BPD requiring tracheostomy often have other morbidities, with one multicenter study noting 58% of infants with a tracheostomy also had BPD-PH, of which 33% required outpatient pulmonary antihypertensive medications [6]. It has been reported that lesions of the large airways are also common in this population, including tracheobronchomalacia (TBM) (40–74%), subglottic stenosis (48%), and airway edema (48%) [6,30]. Concurrent large airway lesions, such as TBM, may result in sometimes dramatic increases in the work of breathing that can delay decannulation [31]. Many patients with concurrent large airway lesions require surgical repair of the airway to achieve successful decannulation [6].

### 2.6. Growth and Feeding

All infants with severe BPD are at high risk for sub-optimal growth, which can impact short- and long-term pulmonary outcomes [29,32]. Intrauterine growth restriction, small for gestational age status, and postnatal undernutrition have all been associated with delayed alveolar development, abnormal lung healing, and reduced postnatal gains in lung function [33]. Alternatively, positive linear growth has been associated with an ability to wean from respiratory support in infants with BPD [34]. Patients who demonstrate catch-up weight gain and linear growth have also been shown to have improved pulmonary function testing in childhood [35,36]. Information about growth-related outcomes in patients with BPD requiring tracheostomy is limited. One single-center study examined growth velocity before and after tracheostomy placement in infants with severe BPD and found stable improvements in weight and length growth by four weeks after the first tracheostomy tube change [29]. In this study cohort, there was no change in pre- and post-operative respiratory severity scores and an overall decrease in caloric intake following tracheostomy [29], leading the authors to speculate that this improving growth may result from reduced stress and work of breathing following tracheostomy. The authors also noted that while this improvement in growth was noted at the four-week mark, many infants continued to have some degree of linear growth failure throughout their hospital stay [29]. Another single-center study examined the growth outcomes following hospital discharge in a general cohort of infants with tracheostomy and found significant improvement in weight and weight-for-length z-scores by six months to one year of age, which continued through three years of age [16]. However, further studies are needed to determine if, and to what extent, tracheostomy impacts linear growth in infants with severe BPD.

Almost all infants with severe BPD and tracheostomy require surgical feeding tube placement [6,7]. Additionally, approximately one-third of infants with tracheostomy also undergo surgical management of gastroesophageal reflux, i.e., Nissen fundoplication, gastrojejunostomy tube, or jejunostomy, although these rates are highly variable and center-specific [6]. Tracheostomy placement may negatively impact oral feeding through changes in swallow mechanics, altered sensation, and changes to the perception and olfaction of food [5]. Dysphagia is common, with one center showing that 80% of patients with tracheostomy experienced symptoms in the outpatient setting [37]. Feeding therapeutic interventions are important aspects of care for all infants and children following tracheostomy placement.

### 2.7. Neurodevelopment

All infants with severe BPD are at risk for neurodevelopmental impairment (NDI) [38], and many factors can impact neurodevelopment in severe BPD. For example, postnatal corticosteroid exposure, particularly dexamethasone, has been shown to increase NDI [39]. Infants with severe BPD are often exposed to prolonged analgesics and sedatives, which have also been shown to negatively affect neurodevelopment. Midazolam has been shown to impact hippocampal development and long-term learning memory [40]. Opioids have been shown to lead to long-term changes in memory and brain function secondary to apoptosis in microglial cells and neurons [40]. Additionally, infants with severe BPD often experience frequent skin-breaking laboratory draws and painful procedures which have been associated with worse neurodevelopmental outcomes in the first two years of life [41]. While all infants with BPD are at risk for NDI, infants with severe BPD and tracheostomy are at the highest risk [22,38]. It is unclear how tracheostomy placement impacts neurodevelopment in patients with severe BPD; it may be a selection bias for those with the severest disease and therefore the greatest exposure to the negative stimuli discussed above, or there may be something intrinsic in having a tracheostomy that negatively impacts neurodevelopment. Although studies are necessary to elucidate the mechanisms associated with the increased risk of NDI in severe BPD patients with tracheostomies, these patients require intensive child development interventions following tracheostomy placement and continuing throughout childhood.

It is unclear if the timing of tracheostomy impacts neurodevelopmental outcomes. Given the importance of oral stimulation on neurodevelopment in infancy, it is reasonable to postulate that earlier tracheostomy, involving taking away the endotracheal tube and fixation devices, may have a positive impact on neurodevelopment in this extremely high-risk group. One retrospective cohort study examined the neurodevelopmental outcomes at 18–22 months of age in former preterm infants who underwent tracheostomy among 16 centers in the NICHD Neonatal Research Network to assess the association of tracheostomy with adverse neurodevelopmental outcomes [22]. The authors found that the adjusted odds ratio (aOR) for the composite outcome of death or NDI in children who received earlier tracheostomy (before 120 days of life) compared with those who received later tracheostomy (after 120 days of life) was 0.5 (95% CI 0.3–0.9). The authors reported that the severity of illness, indication for tracheostomy, and other factors may have influenced the timing of tracheostomy and/or the developmental outcomes; and concluded that further studies are needed to confirm this association before considering earlier tracheostomy in this population [22].

## 3. Tracheostomy Decision Making

### 3.1. Tracheostomy Indications

There are no accepted national standards for tracheostomy indications, and therefore indications for tracheostomy vary from center to center as well as from provider to provider [10]. In most centers, tracheostomy is often considered for infants with severe BPD that require “long-term” IPPV or have structural airway problems that cannot be immediately surgically corrected. However, there are no standard definitions for long-term IPPV or even for structural airway problems. Other potential indications, either alone or in combination, may include infants that cannot be liberated from non-invasive positive pressure support (i.e., nCPAP), have significant growth failure, experience equipment interface difficulties, and/or need a relatively high and on-going supplemental oxygen requirement. Given that there are no standard indications for tracheostomy placement, observational studies have attempted to understand the risk factors for tracheostomy in patients with severe BPD as a first step in identifying phenotypes associated with high-risk of tracheostomy in severe BPD. These risk factors include infants born at a later gestational age, those small for gestational age, and those with BPD-PH [5,42].

### 3.2. Timing of Tracheostomy

The decision to place a tracheostomy is usually very difficult for families and caregivers; this is in part due to fear and anxiety for families and a common feeling among healthcare providers of failure when an infant needs a tracheostomy. Therefore, it is common to delay tracheostomy decision making until well past the diagnosis of severe BPD which is made at 36 weeks PMA, and even well past corrected term age (40 weeks PMA). Another contributing factor to waiting so long to decide on tracheostomy is that it has been reported that mortality and postoperative complications related to tracheostomy are seen more commonly in preterm infants compared to term infants [43]. However, just as there is no consensus on indications for tracheostomy, there is currently no consensus on when the appropriate time for tracheostomy placement. The available literature consists entirely of observational studies and suggests a median age of tracheostomy placement between 43–51 weeks PMA, and that the timing of tracheostomy placement is highly center-dependent (Table 2). One study examining tracheostomy timing among 12 tertiary care centers involved in the BPD Collaborative found a median age of tracheostomy placement of 48 weeks’ PMA or five months chronological age (IQR 4–7 months) [6]. Another study reported a median age at tracheostomy placement of 46 weeks’ PMA (IQR 43–52 weeks’ PMA) among 21 centers in the Children’s Hospitals Neonatal Consortium [10].

The timing of tracheostomy placement often involves waiting for multiple failed attempts at weaning from mechanical ventilation [12,44]. The notion that “we should try one more time” often underlies this and is a deliberate attempt to avoid tracheostomy given the risks for mortality and morbidity as well as the implications for parents and caregivers of a technology dependent infant. Clinicians will often also try to optimize nutrition and lung growth, believing that with enough time this may allow some infants to be successfully liberated from mechanical ventilation without needing a tracheostomy [43]. Other centers choose to proceed with tracheostomy placement to enable engagement in developmentally appropriate activities, decrease the need for pharmacologic sedation, and potentially reduce laryngotracheal stenosis [22]. Ultimately, there is no high-level evidence regarding the best time to recommend and proceed with tracheostomy placement for patients with severe BPD. Clinical studies to generate high-quality evidence are desperately needed to determine when the benefits of tracheostomy placement outweigh the risks of the procedure, anesthesia, and short- and long-term complications.

### 3.3. Family-Centered Care

A family-centered approach that is built on shared decision making is absolutely necessary for all conversations and decisions regarding tracheostomy placement. When counseling families regarding tracheostomy, providing the available information regarding short- and long-term outcomes in an understandable fashion for each family is essential. Determining the most important factors influencing each family’s decision making is also key in arriving at a decision that will provide the very best outcome for a given patient and family. One study found that parents of infants with BPD often prioritize outcomes related to physical health and safety over outcomes related to neurodevelopment. In this study, parents were more concerned about breathing, growth, feeding, and safety outcomes, and were more willing to accept difficulties with learning and behavior [45]. These findings in the families of BPD patients are similar to those in a study that revealed that parents of infants in the neonatal intensive care unit do not view mental and cognitive delay as indicators of an impaired quality of life [46]. When counseling families, it is important to consider each individual family’s concerns and determine what is most important to them regarding short- and long-term outcomes.

There is no question that a tracheostomy will affect family dynamics and family health. For example, in a recent review parent experiences and views related to having a child with a tracheostomy were examined and social isolation was commonly reported, which resulted from many factors, such as worry about leaving home and social stigma [47]. Some studies have reported marital difficulties related to the stress of caring for a child with a tracheostomy, and the parent remaining in the home often feels even more socially isolated [48,49]. Some parents also reported tension, fear, personal strain, and a high psychological burden that can adversely affect health and emotional well-being. Conversely, some parents reported a strong ability to cope with caring for their child with minimal impact on their life. Some of the reported coping strategies included finding ways to sustain everyday routines and environments. A core finding in this review was that parents reported being committed to providing their child with a good quality of life, and parents often rated their child’s quality of life as better than their own [47]. Thus, it is imperative to understand each family’s needs and support system to fully utilize shared decision making around tracheostomy placement.

### 3.4. BPD, Tracheostomy, and Social Determinants of Health

There is a growing body of literature that has examined the effects of race/ethnicity and socioeconomic status on outcomes for infants and children with severe BPD. Infants born to Black mothers have been shown to have an increased likelihood of mortality and an increased length of hospital stay compared to infants born to White mothers [50]. Sociodemographic status measured by neighborhood deprivation index and neighborhood median household income have also been associated with an increased likelihood of mortality and higher rates of readmission in patients with BPD [51,52]. One study examined if certain race/ethnicity and sociodemographic factors were associated with tracheostomy insertion and found that Black infants had 25% higher odds of tracheostomy insertion compared to White infants [13]. Hispanic infants had 20% lower odds of tracheostomy insertion compared to White infants [13]. This study also found that patients receiving public health insurance had increased odds of tracheostomy insertion [13]. These differences were not explained by differences in gestational age at birth or the presence of comorbidities. It is unclear what leads to the disparities in the use of tracheostomy in patients with BPD, and these disparities must be further studied to develop plans to mitigate these health inequalities.

### 3.5. Care Coordination

It is important that patients be at a center that utilizes an interdisciplinary team for severe BPD management when deciding on tracheostomy [53]. Evidence suggests that a multidisciplinary care team can improve survival in infants with severe BPD and tracheostomy [19,42]. Additionally, multidisciplinary discussions with the family before tracheostomy regarding short- and long-term risks, outcomes, prognosis, discharge planning, and outpatient care are essential. Multidisciplinary team members should include the intensive care team, pediatric pulmonology, otolaryngology, pediatric surgery, palliative/supportive care, psychology, nursing, social work, and care management.

Consideration of the long-term outpatient support needed for the infant if the family and team proceed with a tracheostomy is essential. Infants with tracheostomy require multidisciplinary care in the outpatient setting, with co-management by a general clinician and a respiratory subspecialist, such as a pediatric pulmonologist or neonatologist [54]. At least two trained caregivers are needed at home to care for the infant after discharge, one of whom should always be awake and present in the home [54]. Many pieces of equipment will be required, including a home ventilator, a backup ventilator, batteries, a self-inflating bag and mask, a heated humidifier, supplemental oxygen for emergencies, suctioning equipment, and a pulse oximeter [54]. It is ideal to have nursing care support for the family; however, given the current state of home nursing care and the resultant limited availability, it is becoming more and more common for parents and extended family members or friends to carry out this complex caregiver role at home [47].

### 3.6. Making the Decision to Place a Tracheostomy

First and foremost, there is no high-quality evidence on which to base a decision on, or the timing of, for tracheostomy in patients with severe BPD. There is clearly a group of infants with severe BPD who cannot be liberated from invasive positive pressure ventilation, and to facilitate airway, lung, and neurological development at some point a stable and safe airway (i.e., a tracheostomy) is necessary. However, currently reaching that decision can be quite difficult. Obviously, the decision to place a tracheostomy must be a shared decision including the parents of the patient and the various disciplines involved in the patients pre- and post-tracheostomy care. Occasionally, we have had the experience where families push for a tracheostomy; however, it is much more common that parents and/or caregivers want to delay tracheostomy placement and try “just one more time” to extubate the patient. Thus, this decision is often made only after some sort of agreement is reached that everything that has been tried to avoid tracheostomy placement has failed. This may be completely appropriate given that there are substantial risks with tracheostomy, but it may inadvertently cause undue stress for parents and caregivers, and it may make the decision seem arbitrary, subjective, and/or one-sided. To attempt to make this difficult decision at least a bit more objective and include longitudinal assessments, we have developed a tracheostomy scoring tool for our BPD unit that includes risk factors and assesses them longitudinally to monitor trends over time. The risk factors used in this tool are based on our experience and include respiratory factors (prolonged requirement for high or increasing supplemental oxygen, inhaled nitric oxide, and/or anti-pulmonary hypertensive medications), growth factors (sub-optimal growth, especially linear growth, despite good nutrition), neurodevelopmental factors (ability to participate in developmentally appropriate activities), and medication needs (high-dose chronic systemic steroids, multiple neuro-sedative medications, etc.). Trending this tracheostomy score starting at 36 weeks in intubated patients facilitates conversations and family education around the potential for tracheostomy placement. For example, the need for the scoring and how it works are explained to the parents even if the healthcare providers do not think the patient will eventually require tracheostomy. In our experience, this tool has helped with communication related to tracheostomy between parents and healthcare providers and among the multidisciplinary healthcare team. We would encourage centers to develop similar tools (scoring systems, guidelines, or protocols) that allow for longitudinal assessment and that bring at least some objectivity to the decision and timing for tracheostomy placement. Having a center-specific guideline or protocol that includes longitudinal assessments is very helpful for families to understand that a tracheostomy is being considered and what the objective criteria that need to be met are for recommending tracheostomy placement, which helps to alleviate some of the stressors associated with the decision. However, it should be re-iterated that there is currently a lack of standard indications for, and the timing of, tracheostomy placement in patients with severe BPD and that this gap in our knowledge needs to be addressed urgently with studies that provide high-grade evidence. In what follows, we will discuss what happens after the decision has been made and the tracheostomy has been placed.

## 4. Post-Tracheostomy Management

### 4.1. Tracheostomy Care

The initial post-operative period is a time of high risk for complications related to accidental decannulation, with difficulty replacing the tracheostomy tube, development of a false passage, and wound and skin care complications. For this reason, tracheostomy patients are typically monitored closely by the surgical team until the first tracheostomy tube change, usually performed between post-operative days four and seven [55]. Following this initial period, care of the tracheostomy site includes tracheostomy tube changes every two to four weeks, daily tracheostomy tie changes, and frequent skin and stoma care and cleaning. Some patients may intermittently develop so called tracheostomy-associated tracheitis, which is a poorly defined clinical entity that most sources in the literature describe as an increase or change in secretions along with signs of clinical worsening, including fever or the need for increased respiratory support [56]. However, the diagnostic criteria for tracheostomy-associated tracheitis varies between centers and providers. The role of cultures taken via the tracheostomy tube or from the site in identifying and treating pathogenic bacteria is highly controversial, as the colonization of the airway in tracheostomy patients is ubiquitous [56]. On one extreme, some authors have advocated for regular surveillance cultures to monitor colonizing organisms and detect pathogenic alterations in respiratory flora [57]. However, there is a complete lack of evidence in infants with severe BPD and tracheostomy on whether the monitoring of surveillance cultures or the treatment of tracheitis results in any improvement in outcomes [58,59]. Regardless, tracheostomy-associated tracheitis is responsible for a significant burden of readmission, antibiotic treatment, and hospital days [59]. Thus, there is a pressing need for randomized controlled trials to understand what tracheostomy-associated tracheitis is, and how it should be treated.

The development of stomal granulation tissue is another relatively common complication in the post-operative period following tracheostomy placement. Granulation tissue can lead to bleeding, discomfort from the tracheostomy stoma, and difficulty with tracheostomy changes. Topical antibiotic and steroid treatment may help treat early granulation tissue, while persistent granulomas often require cauterization or surgical debridement.

There is no consensus on the role, frequency, and timing of direct laryngoscopy and bronchoscopy (DLB) in the surveillance of tracheostomy patients; however, regular surveillance may help in monitoring the appropriateness of the size of the tracheostomy tube as the patient grows as well as identification and treatment of injury to the airway lumen and proximal obstruction [60]. Lesions in the airway, including glottic or subglottic stenosis, or suprastomal tracheal granuloma or collapse, have been reported in as high as 87% of surveillance DLBs in pediatric tracheostomy patients [61]. These airway lesions may impact both the safety of the patient in the event of tracheostomy plugging or dislodgement and the ability to proceed with decannulation once ventilator support is no longer needed.

### 4.2. Discharge

The American Thoracic Society developed a clinical guideline for infants undergoing tracheostomy, focusing on discharge criteria, caregiver education, and chronic home ventilation needs [54]. At least two trained family caregivers need training and education on caring for the child at home. This training for parents is extensive and includes respiratory status assessment, tracheostomy care, tracheostomy tube changes, suctioning, and how to respond to emergencies such as tube displacement. Additionally, caregivers should receive training on the home ventilator, medication administration, and feeding tube management. Most centers additionally require training in cardiorespiratory resuscitation.

There is variation by center in specific medical indicators for a safe discharge. Consistent respiratory stability, proportional growth, full enteral feeding, and optimization of medications are usually achieved before discharge. There are no guidelines on the maximum acceptable FiO_2_ for discharge, although some centers require an FiO_2_ below 0.40 [62]. As expected, the timing of discharge for infants with tracheostomy also varies by center and depends on considerations for safe discharge at each center, which may include clinical status, caregiver education and training, social determinants of health, and the availability of home nursing services [3]. The disposition following discharge often depends on the family and the available resources. Some infants will be discharged home, while others will be transitioned to long-term chronic care facilities. Most infants are discharged on chronic mechanical ventilation typically with synchronized intermittent mandatory ventilation with pressure support. Some patients are on CPAP or CPAP with pressure support. A minority of infants remain hospitalized until they are stable on tracheostomy collars. The mode of respiratory support at discharge is in large determined by the considerations for safe discharge above.

### 4.3. Outpatient Management

The clinical guidelines for the outpatient management of infants with BPD have become available recently [63,64]. These clinical guidelines suggest long-term monitoring with lung imaging and pulmonary function testing. Commonly used medications such as bronchodilators, steroids, and diuretics are also discussed. There are additional recommendations for the management of home ventilation and supplemental oxygen. These guidelines are based on systematic reviews of the available literature and expert option, and unfortunately the available literature to guide these recommendations is limited with low certainty of evidence [63,64]. This lack of evidence is a major contributing factor to the significant variation of care regarding the outpatient management of infants with severe BPD with tracheostomy [9,54].

There are no clinical guidelines for home ventilator weaning for patients with BPD with tracheostomy, and this process remains highly variable between centers and clinicians. The timing of weaning often depends on the degree of BPD severity and presence of additional morbidities such as BPD-PH or sub-optimal growth. In theory, the placement of tracheostomy tube (which has a larger diameter compared to conventional endotracheal tubes) may be associated with lower resistance; however, to the best of our knowledge there have been no studies examining changes in pulmonary function before and after tracheostomy placement in infants with BPD. While this may be an area for future studies, it is also plausible that readiness for ventilator weaning coincides with improvements in the BPD disease course. Infants and children are often weaned from daytime support before attempting weaning from overnight support [62]. Weaning is usually based on pulse oximetry data, end-tidal carbon dioxide monitoring, frequent clinical assessments, and/or polysomnography. As ventilator technology continues to improve, there may be a future role in utilizing this technology to assist with the development of ventilator weaning algorithms. Additionally, the emerging literature regarding new approaches to ventilator weaning in the adult population may provide guidance for future studies with infants and children with BPD [65].

### 4.4. Considerations for Decannulation

The American Academy of Otolaryngology issued a clinical consensus statement regarding many facets of tracheostomy care to help reduce care variations between clinicians, including the assessment of readiness for and accomplishment of decannulation [66]. Once a patient has been weaned from mechanical ventilation, including during episodes of illness, the status of swallowing and the patency of the airway should be assessed. There should be no documented ongoing aspiration that would necessitate the presence of the tracheostomy tube for pulmonary toilet and secretion clearance. The evaluation of airway patency includes awake flexible laryngoscopy, ideally revealing at least one mobile vocal fold, and micro-direct laryngoscopy to confirm airway patency distal to the glottis. The patient should tolerate capping of the tracheostomy tube all day. Once this is tolerated, patients should undergo either capped overnight polysomnography or a nighttime capping trial in the hospital setting. Finally, if a patient is admitted to the hospital for decannulation and observation on pulse oximetry monitoring for one to two nights before discharge home without the tracheostomy tube, a dressing should be kept over the stoma, and water precautions should continue until stoma closure is confirmed. Up to 65% of patients can have a persistent tracheocutaneous fistula (TCF) six weeks following decannulation [67] and require surgical fistula closure. The factors associated with persistent TCF include younger age at the time of tracheostomy placement and the duration of tracheostomy dependence.

## 5. Future Directions

There are large gaps in our knowledge related to placement of tracheostomy in patients with severe BPD. First and foremost, high-quality evidence must be generated on indications for, and the timing of, tracheostomy placement. Second, high-quality evidence must be generated regarding the maintenance of tracheostomy, including that with regard to the definition, diagnosis, and need for treatment of bacterial tracheitis. Third, studies should continue to provide evidence for ways to optimize neurodevelopmental outcomes in infants and children with tracheostomy, particularly those related to speech and feeding outcomes. Finally, studies should examine whether there are alternatives to tracheostomy that are safe and allow for both pulmonary improvement and optimized neurodevelopment. For example, it has been reported that home CPAP may be used if the infant is able to maintain saturations and remain stable on low flow oxygen for 8 or more hours during the day [68]. Similarly, there are some centers that have tried anecdotal high flow nasal cannula in a very few infants/children with chronic lung disease in an attempt to avoid tracheostomy. There are no reports in the literature regarding the safety or efficacy of these practices to avoid tracheostomy, therefore further studies are necessary prior to their widespread adoption.

## 6. Conclusions

The decision to proceed with tracheostomy comes with significant risks for mortality and morbidity. However, there are currently no available alternatives for long-term invasive or non-invasive positive pressure support outside of the hospital setting. Thus, the decision to place a tracheostomy is often very stressful for both families and caregivers. The development of center-specific guidelines for assessment of need for tracheostomy placement can alleviate some of that stress and result in better shared decision making. However, high-quality evidence is urgently needed to determine the indications and timing for tracheostomy placement and specific risk factors to aid in identifying which patients are at the highest risk for mortality. We must continue questioning our current practice related to tracheostomy placement and strive for better short- and long-term outcomes for this vulnerable population.

## Figures and Tables

**Table 1:** A summary of articles describing morality rates for patients with tracheostomy.

Reference Number	Primary Author (Year)	Cohort	Time Frame Examined	Mortality Rate	Mortality Rate Time Frame
[6]	Manimtim (2023)	155 patients with BPD and tracheostomy from the BPD Collaborative Outpatient Registry at 12 tertiary care centers	2016–2021	2.60%	From initial hospital discharge to the time of review, the median patient age at the time of review was 32 months of age
[13]	Smith (2023)	1614 patients with BPD and tracheostomy at hospitals contributing to the Vizient Clinical Database/Resource Manager	2012–2020	14.10%	
[14]	Akangire (2023)	98 patients with BPD and tracheostomy at one center who survived to initial discharge	2004–2017	1.00%	Post-discharge mortality, data collected up to 4 years of age
[15]	House (2021)	49 patients with BPD and tracheostomy at a single center	2012–2015	26.10%	Data collected until five years of age, 83% died in initial hospitalization
[16]	Akangire (2021)	204 patients with tracheostomy at a single center	2005–2015	21.10%	
[17]	Han (2020)	3442 very low birth weight patients with tracheostomy from 796 North American centers	2006–2016	18.80%	One-year initial hospital mortality rate
[11]	Friesen (2020)	14,155 patients with tracheostomy among 52 children’s hospitals in the United States	2010–2018	8.60%	Initial hospital mortality rate
[18]	Strang (2018)	132 patients with tracheostomy at a single center	2010–2015	14.40%	12-month mortality rate
[19]	Kinsella (2017)	27 patients with BPD and tracheostomy at a single center after the implementation of a ventilator care program	2006–2013	15.00%	Initial hospital mortality rate
[10]	Murthy (2017)	1383 patients with BPD from the Children’s Hospitals Neonatal Database at 21 centers	2010–2013	20.2% for the combined outcome of death or tracheostomy	
[20]	Funamura (2017)	513 patients at one tertiary care hospital with tracheostomy	1984–2015	16.60%	Data collected until up to 18 years of age, 34% died in initial hospitalization
[21]	Watters (2016)	502 patients who underwent tracheostomy placement in 2009 that were enrolled in Medicaid from 10 states	2009	9.00%	First two years following tracheostomy
[22]	DeMauro (2014)	304 patients with tracheostomy and premature birth from the Neonatal Research Network	2001–2011	8.20%	Death after 36 week’s PMA
[12]	Mandy (2013)	22 patients with BPD and tracheostomy at a single center	2004–2009	22.70%	Death before initial hospital discharge

**Table 2:** A summary of articles describing the median age of tracheostomy placement, discontinuation of positive pressure, and age of decannulation.

Reference Number	Author (Year)	The Median Age of Tracheostomy Placement	The Median Age for Discontinuation of Positive Pressure	The Median Age for Decannulation
[6]	Manimtim et al. (2023)	48 weeks’ PMA	27 months	49 months
[14]	Akangire (2023)	43 weeks’ PMA	24 months	32 months
[15]	House (2021)	43 weeks’ PMA	27 months	44 months
[16]	Akangire (2021)	4.5 months	23 months	38 months
[10]	Murthy (2017)	46 weeks PMA		
[7]	Cristea (2013)		24 months	37.5 months
[12]	Mandy (2013)	51 weeks’ PMA		

## Data Availability

No new data were created or analyzed in this study. Data sharing is not applicable to this article.
